# Vinculin Motion Modes Analysis with Elastic Network Model

**DOI:** 10.3390/ijms13010208

**Published:** 2011-12-27

**Authors:** Xiong Jiao, Shan Chang, Lifeng Yang, Meiwen An, Weiyi Chen

**Affiliations:** 1 Institute of Applied Mechanics and Biomedical Engineering, Taiyuan University of Technology, Taiyuan 030024, China; E-Mails: anmw_jx@sina.com (M.A.); chenwy_jx@sina.com (W.C.); 2 College of Informatics, South China Agricultural University, Guangzhou 510642, China; E-Mail: schang@scau.edu.cn; 3 College of Computer Science and Technology, College of Software, Taiyuan University of Technology, Taiyuan 030024, China; E-Mail: yanglifeng@tyut.edu.cn

**Keywords:** vinculin, motion mode, elastic network model, activation mechanism

## Abstract

Vinculin is an important protein for the linkage between adhesion molecules and the actin cytoskeleton. The activation mechanism of vinculin is still controversial. In order to provide useful information for a better understanding of its activation, we analyze the motion mode of vinculin with elastic network model in this work. The results show that, to some extent, the five domains will present structural rigidity in the motion process. The differences between the structure fluctuations of these domains are significant. When vinculin interacted with other partners, the central long alpha-helix of the first domain becomes bent. This bending deformation can weaken the interaction between the first domain and the tail domain. This motion mode of the first domain is in good agreement with the information extracted from some realistic complex structures. With the aid of the anisotropy elastic network mode, we analyze the motion directions of these domains. The fourth domain has a rotational motion. This rotation is favorable for the releasing of the tail domain from the pincer-like clamp, which is formed by the first and the third domain. All these motion modes are an inherent feature of the structure, and these modes mainly depend on the topology character of the structure.

## 1. Introduction

As a cytoskeletal protein, vinculin is associated with cell-cell and cell-matrix junctions through the link between the integrin adhesion molecules and the actin cytoskeleton [1]. Vinculin is very critical for the control of the cytoskeletal mechanics [2], cell spreading and lamellipodia formation [3,4]. Once a shortage of the vinculin has occurred, various functions of the cell will be affected, such as the disruption of the focal adhesion complex [5] and the prevention of cell adhesion and spreading.

**Figure 1:**
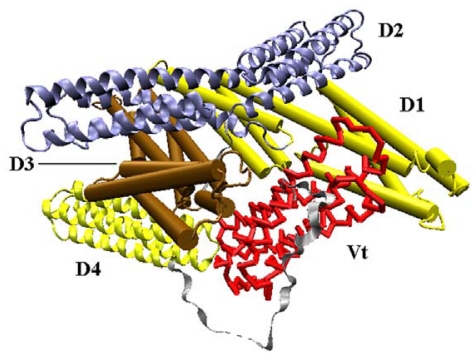
The front view of vinculin (PDB ID: 1TR2). D1 and D3 domain is drawn in cartoon mode, and the D2 and D4 domain is in ribbon mode. The Vt domain is drawn with trace method. D1, D2, D3, and D4 form the head of the protein, and Vt is the tail.

Vinculin contains 1066 amino acids with a 117-kDa molecular weight. As shown in Figure 1, the crystal structure (PDB ID: 1TR2 [6]) of full-length human vinculin contains five distinct domains. These five domains are arranged in an auto-inhibited conformation. Vinculin head (Vh), the N-terminal of the protein, is a globular part, and it contains four domains (D1, D2, D3 and D4). The tail domain of vinculin (Vt), connected to D4 by a proline-rich linker region, is grabbed by a pincer-like structure formed from D1 and D3. The D2 domain can stabilize this pincer-like structure through extensive contacts with D3. For the first three domains (D1, D2 and D3), each of them is comprised of two four-helix bundles, and these bundles are linked by a central long alpha-helix.

Many interaction sites for numerous binding partners have been mapped onto all these regions [7]. The Vh domain contains binding sites for talin and α-actinin, while the Vt domain contains binding sites for F-actin [8] and paxillin [9]. The proline-rich linker region contains binding sites for vinexin [10] and ponsin [11].

In its inactivated state, vinculin holds a “closed” and auto-inhibited conformation. In this inactivated conformation, these binding sites are all blocked by the intra-molecular interaction between the vinculin head and the tail domain. Once vinculin becomes activated, the interaction between Vh and Vt domain will be severed, and all the binding sites will be exposed.

Vinculin activation needs the release of the Vt domain from the binding interaction between the D1 and D3 domains with Vt. However, the detailed structural mechanism of this activation process is still under dispute [12,13]. In some models, it is proposed that vinculin can be activated by a single binding partner [12,14]; while in others models, it is said that vinculin is activated through a combinatorial mechanism that needs at least two binding partners [13,15]. The analysis of this work shows that the binding of D1 with other partners will help the release of Vt, regardless of how many partners are involved in this binding process.

For the activation mechanism of vinculin, experimental research is very challenging. The activation process of vinculin involves a large-scale conformational change, which is related to its biological function [10,16]. Thus, it is difficult to observe the detailed activation mechanism in experiments.

From another perspective, it is very time-consuming to study the conformational change with the molecular dynamic simulation for such a big protein, vinculin [17].

Therefore, it is valuable to do a motion mode analysis for the whole protein with some simple computational models [18]. In this work, we study the motion modes with two simple coarse-grained schemes—Gaussian network model (GNM) [19] and anisotropic network model (ANM) [20,21]. These two models belong to the elastic network model (ENM) [19–26]. The results from these analyses can provide useful information about the motion tendency. This information is helpful in understanding vinculin’s activation mechanism. Moreover, compared with the molecular dynamic simulation, the calculation of the ENM can save a great amount of computer resources.

From the crystal structures, the GNM method can directly estimate the amplitude of the conformation transition. The directional information of this transition can be obtained from the analysis with ANM.

The ENM and normal-mode analysis have been successfully used in biological physics. Such as: the difficult molecular replacement problem [27], the prediction of the opening and closure of member channel [28] and the dynamic allostery controlling of the conformer switching [29,30]. As a simple yet useful tool, GNM method can be used for the large-scale conformational motions, domain motions and collective dynamics of the biomolecular system.

At atomic level, the calculation technique of the low frequency motion modes for a large biological molecule has been developed, and some meaningful works have been carried out [26,31].

## 2. The Gaussian Network Model and the Anisotropy Elastic Network Model

The details of the calculation process of ENM have been described elsewhere [20,32,33], so we present the basic principles of the GNM and ANM.

In the Gaussian network model, the three dimensional structure of protein is described as an elastic network with C-alpha atoms as nodes. These nodes are connected by harmonic springs when two nodes are within a certain cutoff distance (7.3 Å is used in this work). For all springs, the force constant is taken as identical. When we include all contacting residues, the Hamiltonian of the protein system can be defined as follow:

(1) $$V=\frac{1}{2}\gamma [\Delta R^{T}(\Gamma ⊗E)\Delta R]$$

where *γ* is the harmonic force constant. We use N to represent the residues’ number, {Δ*R*} is for the 3N-dimensional column vector for the fluctuation vectors Δ*R*_1_,Δ*R*_2_, ...Δ*R_N_* of these nodes. The superscript T denotes the transposition of a matrix. E is a 3 × 3 identity matrix. Symbol ⊗ represents the direct product. Γ is a N × N symmetric Kirchhoff matrix in which the elements are defined as

(2) $$\Gamma =\{\begin{array}{llll} -1 & \text{if} & i\neq j\;\;\;\text{and} & R_{ij}\leq r_{c}\\ 0 & \text{if} & i\neq j\;\;\;\text{and} & R_{ij}>r_{c}\\ -\sum_{k,k\neq j}\Gamma_{ik} & \text{if} & i=j & \end{array}$$

where *R_ij_* represents the distance between the ith and jth nodes and *r_c_* is the cutoff distance.

The mean-square fluctuations of each node and the cross-correlation fluctuations between different nodes are in proportion to the diagonal and off-diagonal elements of the pseudo inverse of the Kirchhoff matrix. The inverse matrix of the Kirchhoff matrix can be decomposed as:

(3) $$\Gamma^{-1}=U\Lambda^{-1}U^{T}$$

where U is an orthogonal matrix with eigenvectors of Γ as its columns vectors *u_i_* (1 ≤ *i* ≤ *N*). Λ is a diagonal matrix with the eigenvalues of Kirchhoff matrix– λ*_i_* as its elements. The cross-correlation fluctuations between the ith and jth nodes can be calculated by

(4) $$<\Delta R_{i}\cdot \Delta R_{j}>=\frac{3k_{B}T}{\gamma }{\left[\Gamma^{-1}\right]}_{ij}$$

where *k_B_* is Boltzmann constant, *T* is absolute temperature.

When *i* = *j*, we can get the mean-square fluctuations of the ith residue. In the kth mode, the mean-square fluctuation of the ith residue can be calculated with

(5) $${\langle \Delta R_{i}\cdot \Delta R_{i}\rangle }_{k}=\frac{3k_{B}T}{\gamma }\lambda_{k}^{-1}{\left[u_{k}\right]}_{i}{\left[u_{k}\right]}_{i}$$

We do the normalization calculation for the cross-correlation with the follow equation:

(6) $$C_{ij}=\frac{\langle \Delta R_{i}\cdot \Delta R_{j}\rangle }{{\left[\langle \Delta R_{i}^{2}\rangle \cdot \langle \Delta R_{j}^{2}\rangle \right]}^{1/2}}$$

With GNM model, for the fluctuations of nodes, we can only get the information about the amplitudes but no information for the directions. To including the directional information in the analysis process, an anisotropic elastic network model (ANM) is used. In this model, the motion mode of a protein can be determined by the Hessian matrix H.

(7) $$H=\left(\begin{array}{cccc} h_{11} & h_{12} & \cdots & h_{1N}\\ h_{21} & h_{22} & \cdots & h_{2N}\\ \vdots & \vdots & \vdots & \vdots \\ h_{N1} & h_{N2} & \cdots & h_{NN} \end{array}\right)$$

where the element hij is a submatrix with order 3 × 3. The detailed calculation of hij is:

(8) $$h_{ij}=\left(\begin{array}{ccc} \frac{\partial^{2}V}{\partial x_{i}\partial x_{j}} & \frac{\partial^{2}V}{\partial x_{i}\partial y_{j}} & \frac{\partial^{2}V}{\partial x_{i}\partial z_{j}}\\ \frac{\partial^{2}V}{\partial y_{i}\partial x_{j}} & \frac{\partial^{2}V}{\partial y_{i}\partial y_{j}} & \frac{\partial^{2}V}{\partial y_{i}\partial z_{j}}\\ \frac{\partial^{2}V}{\partial z_{i}\partial x_{j}} & \frac{\partial^{2}V}{\partial z_{i}\partial y_{j}} & \frac{\partial^{2}V}{\partial z_{i}\partial z_{j}} \end{array}\right)$$

For the elements of hij, its analytical expression can be written as follows,

(9) $$\{\begin{array}{l} \frac{\partial^{2}V}{\partial x_{i}\partial y_{j}}={\frac{-\gamma (x_{j}-x_{i})(y_{j}-y_{i})}{R_{ij}^{2}}|}_{R_{ij}=R_{ij}^{0}},\text{when }i\neq j\\ \frac{\partial^{2}V}{\partial x_{i}\partial y_{i}}=\gamma \sum_{j\neq i}{\frac{-\gamma (x_{j}-x_{i})(y_{j}-y_{i})}{R_{ij}^{2}}|}_{R_{ij}=R_{ij}^{0}},\text{when }i=j \end{array}$$

The meanings of *γ* and R are the same as in Equation 1. X, y and z represent the position coordinates of atoms.

## 3. The Slow Mode of the Motion

**Figure 2:**
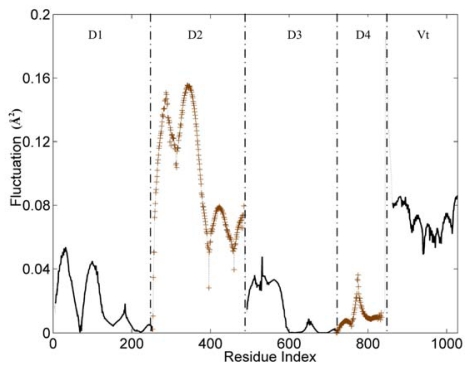
The slowest mode of vinculin structure (PDB ID: 1TR2, chain A). D2 and D4 domains are drawn with ‘+’ mark. D1, D3 and Vt domain are shown with an ordinary line.

The slow and long wavelength collective modes represent the functionally relevant motions of the protein. Figure 2 displays the first mode (the slowest mode) of the whole protein calculated by the GNM, which corresponds to the motion mode with the minimum frequency. The ordinate of this figure represents the normalized distribution of squared fluctuations. For a clear exhibition, the D2 and D4 domains are drawn with ‘+’ mark, and the other three domains are shown with an ordinary line. Along the X axis, the five domains are present in the order of D1, D2, D3, D4 and Vt.

With the slowest mode from GNM, the protein structure can be divided into some dynamic domains, and the hinge sites for the domain motions also can be identified [34–36]. In Figure 2, these five domains can be distinguished clearly by some sharp changes as the boundaries between them. Only for the link between D3 and D4 domain, the change of fluctuation is small. For these five domains, each of them holds different dynamic properties. The hinges between these dynamic domains hold a low fluctuation in this figure.

The whole structure of vinculin is composed of alpha-helix bundles, which has a bigger rigidity than other types of structural elements (such as beta-sheet and loop). Thus, these helixes are distinctly shown as a unit in the figure, especially for the D1 and D2 domain. Each helix corresponds to a line segment. These line segments are separated by the local minima points between them.

For the flexible linker between D4 and Vt domain, it also holds a drastic change of fluctuation. This flexible linker is a little far from the main-body of vinculin, so it will have a small influence on the motion tendency of the whole protein. As a disordered part, this linker is lost in the protein’s structural data. Thus, we did not include this linker in the analysis process. For residues in the tail domain, the residue index is re-indexed simultaneously. The aim of this re-indexing is to repair the index gap due to the lost part.

**Figure 3:**
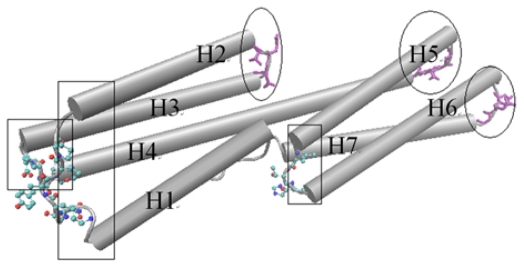
The cartoon diagram for the rotational motion mode of the D1 domain. The regions with low fluctuation are drawn using licorice method, and marked with ovals. The regions with high fluctuation are drawn using CPK method and marked with rectangles. This is a dorsal view of the D1 domain.

D1 domain contains many important binding sites for different binding partners to interact with vinculin. The binding with talin is thought to have an important function for integrin activation and focal adhesion assembly. For the D1 domain, there are three zones that have a low fluctuation. These zones correspond to the link between H (helix) 2–H3, H4–H5 and H6–H7, marked with ovals as shown in Figure 3. As a comparison, there also are some regions with a high fluctuation. These regions include the link between H1–H2, H3–H4 and H5–H6, which are marked with a rectangle in Figure 3. The seven alpha-helixes of D1 can be divided into two parts. One contains H1, H2, H3 and the first half of H4 and the other contains H5, H6, H7 and the second half of H4. As the central helix, H4 is the longest one of these seven helixes.

For a rigid body, if one end maintains a stationary state and the other has a motion tendency, then this rigid body may have a rotational motion, especially when there is an angle between the motion direction with the axis of the rigid body. As shown in Figure 3, the left part of D1 holds high fluctuation in the slow motion mode. At the same time, the opposite end of this part holds low fluctuation. With this information, and considering the complex structures of vinculin D1 with other partners, we can deduce that the left part of D1 will have a rotational motion with the centre of D1 as the axis. The H5 and H6 will also have a rotational motion for the same reason. But in this case, the axis is the link between H4–H5 and the link between H6–H7. In other words, except H4, the other six helixes will have a rotational motion with one of their ends as the axis. As a longer helix is more flexible than a shorter helix, H4 will be bent in this motion process. When these rotations have occurred, the conformation change will facilitate the exposure of the blocked binding sites.

## 4. Cross-Correlations between Atomic Fluctuations

We calculated the cross-correlations between the fluctuations of C-alpha atoms. The modes with low frequency correspond to functional slow motions, and modes with high frequency correspond to localized fast motions. To improve the signal-to-noise ratio, only low frequency modes are used in the calculation.

**Figure 4:**
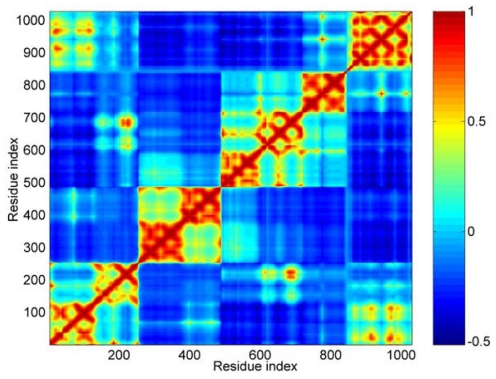
The cross-correlation maps calculated using the first 40 modes. Color ‘blue’ represents negative correlation and ‘orange-red’ positive correlation, as shown in the color bar on the right. Both the axis (x and y) of this map are residue indices.

Concretely, the first 40 modes were used in this work. In Figure 4, the calculated result of cross-correlations is shown as a color map. The cross-correlation value ranges from −1 to 1. A positive value represents the residues move in the same direction, and a negative value represents that they move in the opposite direction. The bigger the absolute value of cross-correlation, the better the two residues correlated (or anticorrelated). Uncorrelated fluctuations correspond to a correlation value of zero. As shown in Figure 4, the blue regions represent the negative correlation, and the orange-red regions are for positive correlations.

For this cross-correlation map, it is obvious that there are four orange-red portions anti-diagonally. These four orange-red portions correspond to the five distinct domains. The boundary between D3 and D4 is not as clear as other boundaries, such as between D1 and D2. For D1, D2 and D3, all three domains are composed of two four-helix bundles. From the cross-correlations map, we can see that these three domains are obviously divided into two parts. These parts correspond to the two helix bundles in each domain, especially for D2 domain. This means that the local structures of these two bundles are more stable than that of the central helix. In other words, the conformation change of these three domains can be shown mainly as the structural change of their central helix, such as bending.

Along the main diagonal section, there are two obvious bright areas. One corresponds to the interactions between the first helix bundle of D1 and Vt. The other represents the interactions between the second bundle of D1 and D3 domain.

As for the D4 and Vt domain, the interactions between them are mainly negatively correlated. But there are still some sites, shown as small bright points in this figure, with positive correlation between them. The correlation between the residue’s fluctuations is dependent on the interaction between them. A strong attraction will lead to a positive correlation motion. This phenomenon means that these two domains (D4 and Vt) will have a tendency to move in an uncorrelated manner, but there are still some weak interactions between some specific regions. The correlation between D3 and Vt is negative. When D4 is removed from the main-body of the molecular structure, the weak interactions between D4 and Vt is helpful for the release of Vt from the crab pincer, which is formed by D1 and D3.

## 5. ANM Analysis for the Motion Direction

In the analysis process for the large-scale motions of nodes, the GNM model can only provide the magnitudes of the displacements departing from their equilibrium positions. In order to get the directions of these displacements, ANM is used to analyze the motion mode of this structure.

**Figure 5:**
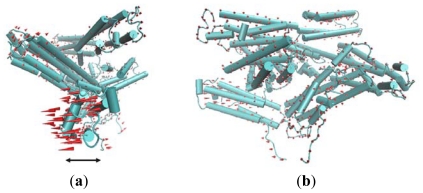
The first slowest motion mode from ANM analysis. The motion modes are presented using the cone model. The length of the cone is correlative with the motion magnitude, and the motion direction is depicted with the orientation of the arrow below the figure. (**a**) The side view of the molecular structure, in which the direction of cones is parallel with this presenting plane; (**b**) The front view of the structure.

The first slowest mode of ANM results mainly corresponds to a rotational motion of D4. In Figure 5, red cones are used to represent this motion tendency. In this motion mode, the N-terminal of D4 has a tendency to rotate away from Vt. For the rigidity of structure, the C-terminal of V4 domain will also have a rotational motion. The N-terminal of D4 is far away from Vt, and the C-terminal of D4 is near to Vt. Thus, when this rotational motion occurs, a passageway will be open for Vt to be released from the pincer-like binding. Then, Vt will become more free. Further, with the help of other partners, the whole protein will become activated.

The second slowest mode from ANM also can be described as a rotational motion involving two domains D2 and D1. For D1, such a rotational motion is also helpful for the release of Vt.

## 6. Motion Tendency Suggested from Complex Structure

When vinculin interacts with different partners, it undergoes a conformational change. This structural change is partners specific. Thus, the information for the real motion of vinculin will be stored in the complex structure, then we can extract this information from the protein structural data.

**Figure 6:**
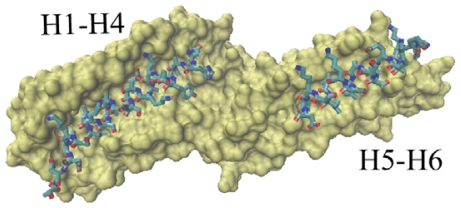
The groove of the vinculin D1 domain bound to other partners. The D1 domain is shown with surf method. The partner is drawn with licorice method. The left part corresponds to the groove formed with H1, H2, H3 and H4. The right groove is formed with H5 and H6. This is a dorsal view of D1 domain.

Figure 6 is a dorsal view of the D1 domain. In other words, Figure 6 observes the D1 domain from the back of Figure 1.

At present, the available vinculin complex structures in PDB are mainly focused on the D1 domain. There are two potential binding regions for this domain. One is the groove formed with H1, H2, H3 and H4, shown in Figure 6 as the left part. The second binding region is a relatively small groove formed with H5 and H6, shown in Figure 6 as the right part.

Correspondingly, the complexes that vinculin D1 interacted with different partners can be classified into two groups. For the first group, vinculin D1 domain interacted with other proteins only at one binding region—the groove from H1–H4 (e.g., 1RKC [12], the complex of vinculin with talin’s vinculin binding site 3). The second group is for the complex in which vinculin D1 interacted with other partners at these two binding regions. For instance, the complex of vinculin interacted with two VBs (vinculin binding sites) of Shigella flexneri’s IpaA (PDB ID: 2IBF [37]).

**Figure 7:**
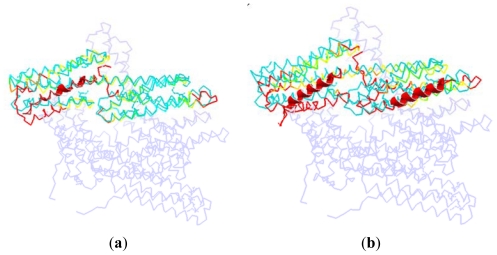
The dorsal view of the complex structure that vinculin is bound to other partners. (**a**) The D1 domain interacted with other partner at only one binding site, (take protein 1RKC as an illustration); (**b**) The D1 domain interacted with other partners at two binding sites (take protein 2IBF as an illustration).

As shown in Figure 7, we superpose the complex structure (1RKC, 2IBF) onto the monomer structure. In this figure, the D1 in the monomer is colored with cyan, and the rest part of the monomer is drawn with light blue. The partner in the complex structure is drawn with cartoon method, which is shown as the helix with red color in this figure. For the D1 domain in the complex, we use different colors to represent different RMSD between these two structures. The red is present for a high RMSD value, and the green is for a low RMSD, and the yellow is for a moderate RMSD.

In the complex of 1RKC, H4 is obviously bent. The external terminal of the helix bundle (H1, H2, H3 and H4) has a rotation due to the attraction from VBs. At the opposite end, the flexible link between H6–H7 has a local conformation change. As there is no binding partner, this change is only due to the flexibility of the linker.

For 2IBF, there are two binding regions. The conformation change of the groove formed with H1–H4 is similar to that of 1RKC. In the other groove, there is also a rotation around the linker between H4–H5, H6–H7. The deformation of the first groove in 2IBF is bigger than that of the 1RKC. This is due to the fact that there are more binding regions in 2IBF than in 1RKC. More binding regions will lead to more interactions between D1 and other partners. Furthermore, more interactions will bring about a bigger conformational change. For the same reason, the conformational change of the linker between D1 and D2 in 2IBF is bigger than that of 1RKC.

In these two situations, the angle of the bent H4 of D1 is opposite to the Vt domain. The position of the middle part of H4 helix remains unchanged, but the two terminals of this helix depart from their equilibrium position and move away from Vt domain. As mentioned above, in conjunction with the interaction between different partners with D1, the conformation of D1 will change greatly. The bending of H4 will weaken the interaction between D1 and Vt. This conformation change is helpful for the release of Vt. Thus, we can say that the binding of D1 with other partners, regardless of whether it interacts at one or two regions, will help D1 to move away from the Vt. Further, through the weakening of the pincer-like structure, this departure will assist the release of Vt.

## 7. Conclusions

The motion modes are inherently dependent on the topology character of the structure. From the analyses mentioned above, we have attained an outline of the motion modes of vinculin. First, the fourth domain rotates around the terminal which is situated away from the tail domain. With this rotation, the pincer-like binding will be weakened creating a passageway for Vt. These factors combine to release the tail domain from the pincer-like binding of D1 and D3. Second, when D1 domain binds with other partners, the longest helix in the first domain becomes bent. This bending is also helpful for the release of the tail domain. Briefly, the rotation of D4 and the bending of H4 of D1 all assist the activation of vinculin. Detailed structural mechanisms of vinculin activation remain unsolved by this experiment. However, this useful information about motion modes, achieved from ENM, improves the understanding of the activation process for vinculin.
